# Francesco Bossa (1941–2019)

**DOI:** 10.1038/s41419-019-1944-z

**Published:** 2019-09-20

**Authors:** Alessandro Finazzi-Agro’

**Affiliations:** Professor of Biochemistry, Department of Experimental Medicine, University of Tor Vergata, 00133 Rome, Italy



The last May 29, Francesco Bossa after a long, invalidating illness, finally rejoined his beloved spouse, Donatella Barra (1941–2014), who passed away four years before, leaving an unfillable void in the roman biochemical community. As a matter of fact, the Bossas were for almost 50 years a point of reference for the investigation of protein structure. Francesco and Donatella met together as students in the well-renowned Istituto di Chimica Biologica at the University of Rome, established and run by Alessandro Rossi Fanelli, who first isolated human myoglobin and was one of the most important investigator in the field of haemoproteins. Rossi Fanelli gathered a flock of brilliant young scientists, who in turn attracted more people interested in the functioning of macromolecules. Among them Paolo Fasella was investigating the function of pyridoxal phosphate containing enzymes and was the mentor of the medical freshman Francesco. Another fellow was Paolo Cerletti, son of Ugo Cerletti, who first introduced electroshock in the treatment of neuropsychiatric patients. Paolo Cerletti was interested in the mitochondrial enzyme succinate dehydrogenase and, to this purpose, gathered a large group of young investigators among whom the Biology student Donatella^1^. Therefore Francesco and Donatella were working side by side from the sixties of the past century. The interaction between the two became more tight when Cerletti left Rome to get a chair first in Camerino and then in Milan cutting down most of the previous collaborations. Then Donatella joined the Fasella’s group starting her lifelong job on peptides and proteins primary structure and the collaboration with Francesco^2^. Thanks to the ability of Rossi Fanelli in finding research funds and his open-minded attitude, Donatella Barra was perhaps the first researcher in Italy to work with a Stein–Moore automatic amino acid analyser. This opportunity gave a burst to everyone in the Istituto as the main goal of most research groups was indeed the study of various kinds of proteins and enzymes. In the main time, Francesco started his brilliant career in the field of Vitamin B6 dependent aminotransferases^3^ a subject that he cultivated for the next 40 years thus gaining a leading role in the field, producing more than 170 peer-reviewed papers. The couple were working together their life long, but keeping well different the respective expertise, Francesco evolving his interest from the experimental study of structure-functions of enzymes to the bioinformatics approach^4^. Donatella had a more varied field of research since she was collaborating with many scientists at home and abroad on the primary structure of a wide array of proteins. In the last years, she was investigating the structural basis of the antimicrobial peptides from frogs skin^5^.

Both of them achieved important academic roles at the University of Rome La Sapienza. Francesco was full professor of Biochemistry and Dean of the Faculty of Sciences. Donatella also became full professor of Biochemistry but at the Medical Faculty and Director of the newly established Department of Biochemistry rightfully entitled to Alessandro Rossi Fanelli.

They both were able to recruit a number of brilliant co-workers that are carrying on with profit in their fields of research.

The Bossa–Barra will be remembered, and not only in Italy, due to the vast array of international collaborations with eminent scientist and to the impact of their papers, as one of the married couple of scientists more influential in the Biochemistry community.

Ciao Francesco and Donatella!
